# Keratin 18 phosphorylation as a progression marker of chronic hepatitis B

**DOI:** 10.1186/1743-422X-7-70

**Published:** 2010-03-24

**Authors:** Ying Shi, Shihui Sun, Yali Liu, Junfeng Li, Tong Zhang, Hao Wu, Xinyue Chen, Dexi Chen, Yusen Zhou

**Affiliations:** 1 State Key Laboratory of Pathogen and Biosecurity, Beijing Institute of Microbiology and Epidemiology, Beijing, China; 2 Department of Infectious Diseases, Capital University of Medical Sciences, Beijing Youan Hospital, Beijing, China

## Abstract

**Background:**

The intermediate filament proteins keratins 18 (K18) and 8 (K8) polymerize to form the cytoskeletal network in the mature hepatocytes. It has been shown that the phosphorylation of K18 at two serine residues, 33 and 52, correlates with the progression of hepatitis C, but little is known of chronic hepatitis B (CHB). In this study, we examined K18 phosphorylation in relation to CHB.

**Results:**

Site-specific phosphorylation of K18 was determined in livers of twelve healthy donors, and non-cirrhosis (n = 40) and cirrhosis (n = 21) patients. On average, progressively higher level of Ser52 phosphorylation was observed in non-cirrhotic and cirrhotic livers, while elevated Ser33 phosphorylation was detected in both livers but no significant difference. Progressive increase of Ser33 and Ser52 phosphorylation correlated with the elevation of both histological lesions and enzymatic activities of alanine aminotransferase in non-cirrhotic livers. In the hepatocytes of an inactive HBV carrier, strong signals of Ser33 phosphorylation were co-localized with viral infection, while only basal level of Ser52 phosphorylation was detected in infected cells.

**Conclusion:**

Assuming all obtained data, our data suggest that K18 phosphorylation is a progression marker for CHB.

## Background

Keratin 18 (K18) is a member of the intermediate filament family comprising ~70 cytoskeleton proteins. Adult hepatocytes contain only K18 and keratin 8 (K8), heteropolymerized to form the filament networks that protect the cells from various mechanical stresses [1-3]. Serious hepatocellular injures usually result in damages in the filament scaffolds. For example, during apoptosis K18 is cleaved into small fragments by caspases. As a suitable indicative of hepatocytic apoptosis *in vivo*, one of the K18 proteolytic fragments, termed tissue polypeptide-specific antigens (TPS), can be readily detected in the plasmas of patients suffering from alcoholic hepatitis [4], nonalcoholic steatohepatitis[5,6], chronic cholecystitis [7] and chronic hepatitis B (CHB) [8]. In addition to scaffolding function, keratin filaments form complex signaling platforms and interact with kinases, adaptors and apoptotic proteins. K18 is involved in modulating hepatocytic apoptosis induced by Fas/TNF family receptors [9-11]. It has been shown that it attenuates TNF-induced cytotoxicity by sequestering the TNF receptor type 1-associated death domain (TRADD) protein from its interaction with the TNF receptor-1 (TNFR1) [12]. K18 is also important for other cellular processes such as mitosis [13], cell cycle progression [14] and responses to stresses [15].

K18 is modified post-translationally at multiple amino acid residues. Many of these modifications are implicated in the functions other than scaffolding. It has been shown that phosphorylation of K18 is most important for its functions in several processes and likely plays a role in liver diseases. For example, the K18 phosphorylation at Ser33 regulates keratin filament organization and modulates its binding to 14-3-3 protein, which in turn, regulates nuclear 14-3-3 redistribution during mitosis and may play a role in hepatocyte mitotic progression [13,16]. On the other hand, the phosphorylation at Ser52 could be involved in protecting hepatocytes from toxin- and stress-induced liver injuries [15]. Furthermore, hyperphosphorylation of K18 is associated with the human liver diseases. Site-specific K18 hyperphosphorylation was shown to strongly correlate with the progression of liver diseases in patients with chronic noncirrhotic hepatitis C virus (HCV) [17]. Mallory-Denk bodies (MDBs), hepatic inclusion bodies observed in diverse chronic liver diseases such as alcoholic and non-alcoholic steatohepatitis, chronic cholestasis, metabolic disorders and hepatocellular neoplasms, are composed of aggregates of, in addition to other proteins, K18 and K8 in disproportional ratio that are hyperphosphorylated at multiple residues in both keratins, including Ser33 and Ser52 in the former, and Ser73 and Ser 341 in the latter [18-22].

The pathogenesis of viral hepatitis is complex and has been attributed to many factors. Possible mechanisms of chronicity in HCV include failure of the immune responses to viral infection that results in inappropriate or ineffective induction of cytotoxic T lymphocytes (CTLs) and production of cytokines. They may lead to continued viral replication, non-specific inflammatory response and fibrosis [23]. Dysregulation of proliferative and apoptotic pathways represents a pro-tumorigenic principle in human hepatocarcinogenesis. Down-regulation of FAS/FAS-L has been detected in patients with chronic hepatitis caused by HCV, suggesting a role of FAS-mediated hepatocytic apoptosis in eliminating infected cells [24]. In the case of primary hepatocellular carcinoma (HCC), integration of the X gene (HBx) of hepatitis B virus (HBV) into the host genome and the ability of the mutant HBx protein to bind to p53 and abrogate p53-mediated apoptosis are implicated in the etiology and molecular pathogenesis [25,26].

The involvement of hepatocytic keratins in apoptosis and other signal pathways prompted us to examine the potential role of K18 phosphorylation in the progression of HBV chronic hepatitis and/or the severity of liver histological lesions. In this report, we evaluated the phosphorylation of K18 in liver tissues chronically infected with HBV, and determined their enzymatic activities of aspartate (AST) and alanine (ALT) aminotransferases, and histological lesions. Our results indicate that K18 phosphorylation at Ser33 and Ser52 may serve as reliable markers for progression of chronic hepatitis B.

## Results

### K18 phosphorylation is a marker of progression of chronichepatitis B

**Figure 1:**
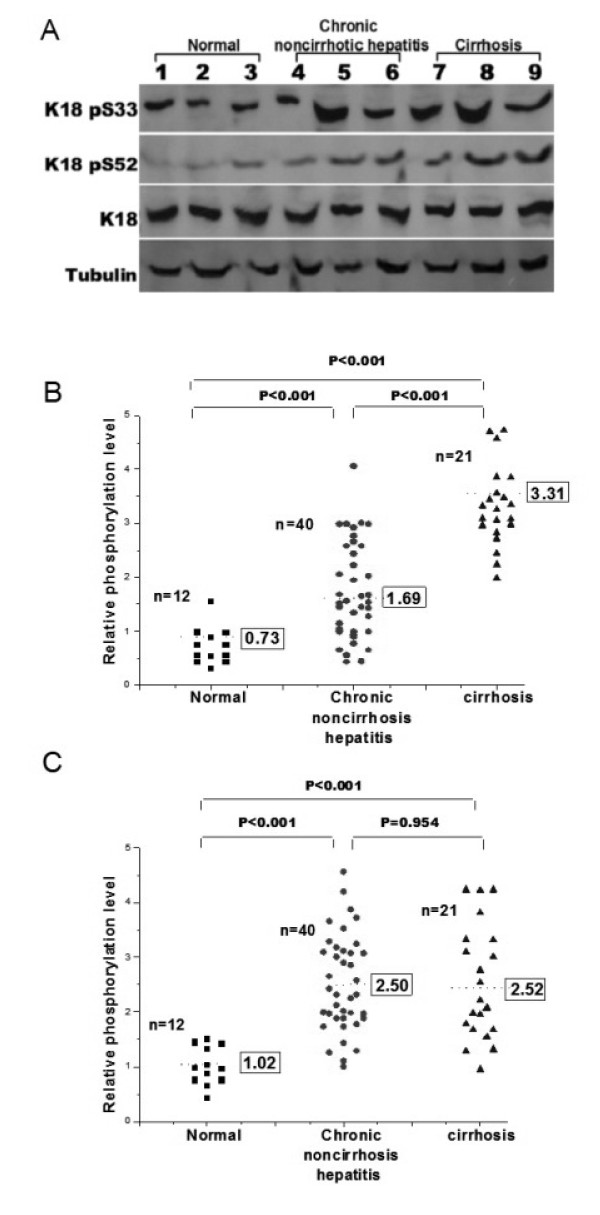
**K18 Phosphorylation in normal, chronic non-cirrhotic hepatitis and cirrhosis livers**. (A) Representative immunoblots of tubulin, K18, Ser33 and Ser52 phosphorylated K18 (K18 pSer33 and K18 pSer52, respectively) from livers of indicated sources. (B) Relative levels of Ser33 phosphorylated K18 from livers of healthy donors (squares), and patients with chronic non-cirrhotic hepatitis (circles) and cirrhosis (triangles). The intensity of K18 of each sample was used to normalize that of K18 pSer33, with the average of each group is shown in the box. The P-values of pair-wise comparisons are indicated on the top. (C) Relative levels of Ser52 phosphorylated K18 from livers of indicated sources, with symbols as the same in B. The P-values are indicated on the top.

HBV infection leads to various clinical presentations, ranging from an inactive carrier state to self-limited acute or chronic hepatitis with progression to cirrhosis and HCC. In this study, we examined the K18 phosphorylation in normal livers and those with chronic non-cirrhotic hepatitis and cirrhosis (Fig. 1A). Liver homogenates containing equal amount of proteins were analyzed by immunoblots probed with antibodies against phosphorylated serine at specific serine residues in K18, and the relative levels of phosphorylation were quantified. While the levels of Ser52 phosphorylation in the normal livers (n = 12) were relatively low with minor fluctuations, those in the chronic non-cirrhotic hepatitis (n = 40) varied in a greater range with an average that was significantly higher than the normal controls (*P *< 0.001) (Fig. 1B). In cirrhotic livers (n = 21), increased levels of Ser52 phosphorylation were detected, with the average significantly higher than the normal (*P *< 0.001) and the chronic non-cirrhotic hepatitis livers (*P *< 0.001) (Fig. 1B). The range of Ser33 phosphorylation in the normal livers was similar to those of Ser52 (Fig. 1C). Significant increases were observed in both chronic non-cirrhotic hepatitis (*P *< 0.001) and cirrhotic (*P *< 0.001) specimens. However, there was no significant difference in the levels of Ser33 phosphorylation between the latter two groups (*P *= 0.954) (Fig. 1C). These results suggest that on average Ser52 phosphorylation increases with the progression of hepatitis B, and that Ser33 phosphorylation increases in hepatitis B, but remains largely unchanged with the progression of the disease.

**Figure 2:**
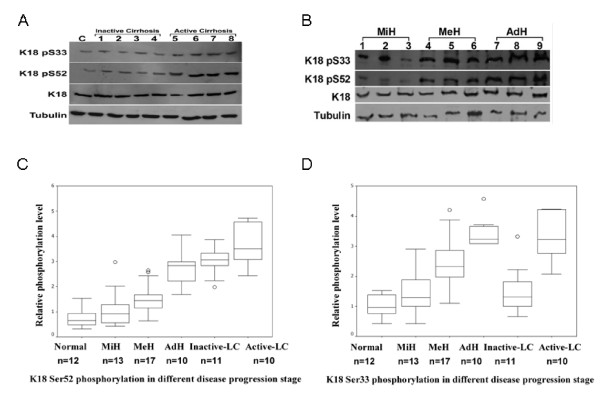
**K18 phosphorylation in relation to histological progression of CHB**. (A) Representative immunoblots of indicated proteins from a control (lane C), inactive (lanes 1-4) and active (lanes 5-8) cirrhotic livers. (B) Representative immunoblots of indicated proteins from livers with progressive histological lesions, MiH (lanes 1-3), MeH (lanes 4-6) and AdH (lanes 7-9), minimal, medium and advanced histological lesions, respectively. (C) The box and whisker plot of the relative levels of Ser33 phosphorylation of K18 in livers of the normal donors, and patients with CHB at different progression stages. Three outliers are indicated by open dots. Significant difference (*P *< 0.005) was observed in any pair with the exception of MiH vs. inactive LC (liver cirrhosis, *P *= 0.750), and AdH vs. active LC (*P *= 0.715), respectively. (D) The box whisker plot of relative levels of Ser52 phosphorylation of K18 in livers from indicated sources. The four outliers are indicated by open dots. Significant difference (*P *< 0.05) was observed in any pair with the exception of normal vs. MiH (*P *= 0.168), and AdH vs. inactive LC (*P *= 0.289), respectively.

However, much deviation and overlapping of K18 phosphorylation was observed in the two groups of the diseased livers (Figs. 1B and 1C). We then asked the question whether there was any correlation between K18 phosphorylation and subgroups or progression of liver lesions within the same group. The 21 cirrhotic specimens were classified to two groups, active (active LC, n = 11) and inactive (inactive LC, n = 10) liver cirrhosis (Fig. 2A)[27]. We divided the 41 chronic non-cirrhotic hepatitis livers to three groups according to Ishak's staging classification [28]: minimal histological lesions (MiH, n = 13), with grading score < 4 and/or staging score < 2; medium lesions (MeH, n = 17), with 4 ≤ grading scores < 8 and 2 ≤ staging score < 3; and advanced lesions (AdH, n = 10), with two scores greater than 8 and 3, respectively (Fig. 2B). The same set of normal livers (n = 12) was included as controls.

The mean relative levels of Ser33 phosphorylation were 1.015, 1.710, 2.500, 3.430, 1.790 and 3.330 for normal, MiH, MeH, AdH, inactive LC and active LC subgroups, respectively (Fig. 2D). Significant differences (*P *< 0.005) were observed in any pair of these six subgroups, with the exceptions of MiH vs. inactive-LC (*P *= 0.750) and AdH vs. active-LC (*P *= 0.715). Similarly, the mean relative levels of Ser52 phosphorylation in the six groups mentioned above were determined to be 0.730, 1.078, 1.556, 2.712, 3.003, and 3.644, respectively (Fig. 2C). Significant differences (*P *< 0.05) were noted in any pair, with the exception of normal vs. MiH (*P *= 0.168) and AdH vs. inactive-LC (*P *= 0.289). These results indicate an overall increase in K18 phosphorylation at both Ser33 and Ser52 with the progression of CHB. There are however minor differences between these phosphorylation events, as the relative level of Ser33 phosphorylation follows the trend of normal < MiH ≈ inactive LC < MeH < AdH ≈ active LC, and that of Ser52 normal < MiH < MeH < AdH < inactive LC < active LC.

### K18 Ser52 phosphorylation is a marker of liver injury, and Ser33 phosphorylation is related to HBV infection

**Figure 3:**
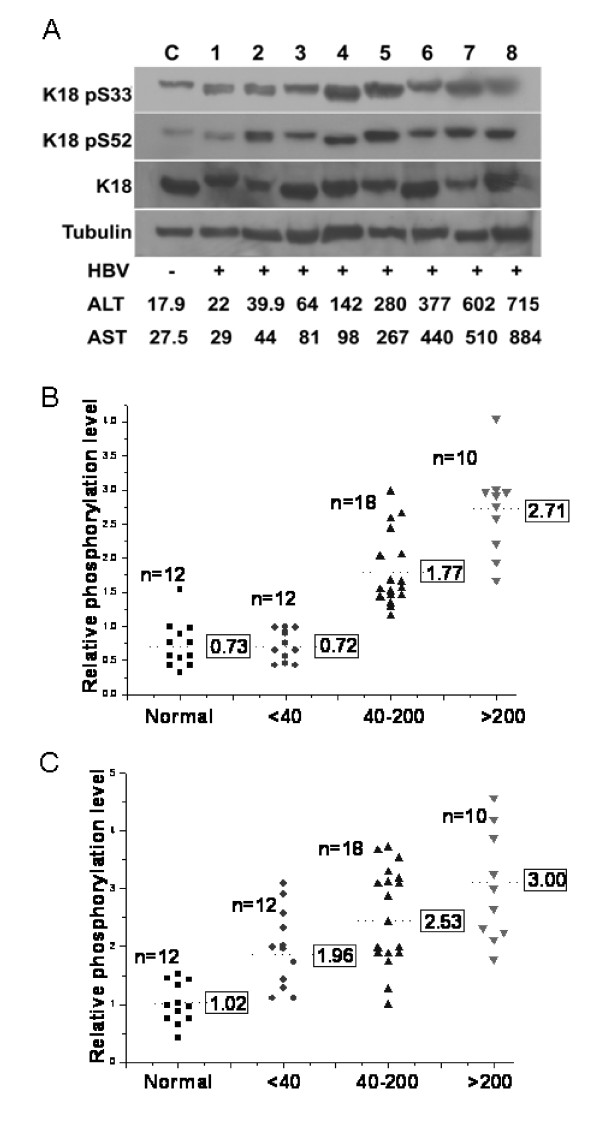
**The phosphorylation of Ser33 and Ser52 in K18 in relation to HBV infection**. (A) Representative immunoblots of indicated proteins from livers of a healthy donor (C), and patients (lanes 1-8) with HBV, and with enzymatic activities of aspartate (AST) and alanine (ALT) aminotransferases (U/L) listed under the each sample. (B, C) Relative levels of K18 phosphorylation at Ser33 (B) and Ser52 (C) in livers of healthy individuals (normal) and HBV patients with ALT (U/L) at low (< 40), medium (40-200) and high (> 200) levels.

Many biochemical and serological markers have been used to evaluate liver injuries. Among these, liver functions (e.g., ALT and AST) are most commonly used. We tested 40 CHB livers for their ALT activities and divided them to 3 subgroups: A, ALT < 40 U/L (n = 12); B, 40 ≤ ALT < 200 U/L (n = 18); and C, ALT = 200 U/L (n = 10). We then compared K18 phosphorylation in these three groups and the 12 normal livers (Fig. 3A). Ser52 phosphorylation was equivalent in the control and subgroup A (*P *= 0.976), while significant increase was noted between subgroups A and B (*P *< 0.001), and subgroups B and C (*P *< 0.001) (Fig. 3B). The positive correlation between levels of phosphorylation and ALT activities suggests that the phosphorylation of Ser52 in K18 is progressively related to liver injuries. On the other hand, significant increase in Ser33 phosphorylation was detected in group A compared with the control (*P *< 0.05). This increase could be an indicative of HBV infection, in addition to a marker of liver injuries. Incremental increases in Ser33 phosphorylation were also observed between subgroups A and B (*P *< 0.05), and B and C (*P *= 0.104), respectively.

**Figure 4:**
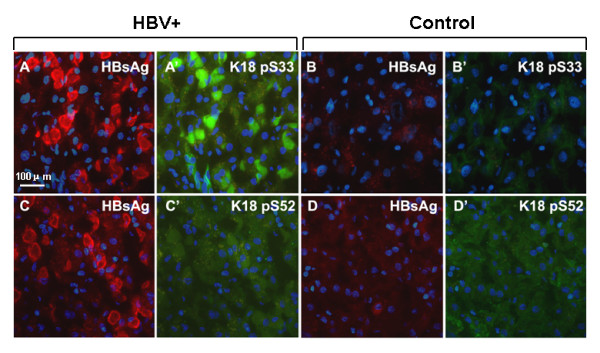
**Detection of K18 phosphorylation and HBsAg in livers from an inactive HBV carrier (HBV+) and a healthy donor (Control)**. Sections of livers were probed for HBsAg (red, A, B, C and D) and K18 phosphorylation (green, A', B', C' and D') at Ser33 (K18 pS33) or Ser52 (K18 pS52), with nuclei stained with DAPI (blue). Representative images from same visual fields (A and A', B and B', C and C', D and D') were selected, with scale bar = 100 μM.

Since direct comparisons between Ser33 phosphorylation in K18 and HBV infection at a cytomorphological level have not been reported frequently, we sought to determine if there was any correlation. A liver specimen respectively from a randomly selected inactive HBV carrier and a healthy donor was sectioned, and probed for HBsAg and phosphorylated Ser33 or Ser52. In the section of HBV-infected patient maintaining normal liver functions, strong signals of phosphorylated Ser33 were restricted to hepatocytes with strong positive detection of HBsAg (Figs. 4A and 4A'). However, only basal levels of Ser33 phosphorylation were detected in the control (Figs. 4C and 4C'), while basal levels of Ser52 phosphorylation were observed in both HBV positive (Figs. 4B and 4B') and healthy (Figs. 4D and 4D') samples. These results reiterate the conclusion that phosphorylation at Ser33 in K18 is reflective of HBV infection.

## Discussion

Posttranslational modifications, e.g., phosphorylation, glycosylation, acetylation, methylation and ubiquitination, play important roles in functional modulation of the intermediate filament proteins. In particular, site-specific phosphorylation of keratins 8 and 18 is involved in regulating the keratin filament structure, interactions with other proteins and other cellular processes [16,29]. Abnormality and hyperphosphorylation of keratins is associated with a variety of liver injuries and diseases. For example, large aggregates of misfolded hyperphosphorylated K8/K18, in disproportional ratio, were found in MDBs of alcoholic hepatitis in both humans and mice [18]. The correlation between keratin phosphorylation and hepatitis C led to the suggestion that the former is a progression marker of the latter when increased [17]. It is therefore crucial to investigate whether there is similar correlation between K18 phosphorylation and CHB caused by viral infection, as it is the most predominant cause of liver disease in China.

We examined liver specimens of 61 patients (40 with chronic non-cirrhotic hepatitis, 21 with cirrhosis) and 12 normal controls. We found that on average Ser52 phosphorylation increased with the progression of CHB, and reached the highest level in cirrhotic livers (Fig. 1C), indicating that it is a progression marker of CHB, as in the case of hepatitis C. Higher levels of Ser33 phosphorylation were also observed in both chronic non-cirrhotic hepatitis and cirrhosis compared with the controls. However, the difference between the two was statistically negligible (Fig. 1B). An earlier study indicated that Ser33 phosphorylation was higher in HCV non-cirrhotic livers than in those of cirrhosis [17]. This discrepancy is likely due to the difference between HBV and HCV hepatitis (see below). These results prompted us to examine the level of K18 phosphorylation in chronic non-cirrhotic livers at different stages as determined by characteristic histological changes, and active and inactive cirrhotic livers (Fig. 2). Although overlapping was observed, Ser52 phosphorylation in general increased progressively with inflammation level, and the average level was higher in active than non-active cirrhosis (Fig. 2D). It is thus evident that Ser52 phosphorylation reflects largely the chronic progression of liver diseases. Ser33 phosphorylation in the inactive cirrhotic livers, on the other hand, was significantly lower than in the active ones. It was also lower than that in the non-cirrhotic livers with higher levels of inflammation (i.e.; MeH and AdH histological subgroups) (Fig. 2C). The level of phosphorylation at Ser33 in K18 seems to correlate with the extent of inflammation. Our results are in consistent with the known implication of Ser33 phosphorylation in resistance to the Fas-mediated apoptosis [10] and sequestering the TRADD protein involved in induction of apoptosis [12], and protective function of Ser52 phosphorylation against hepatotoxic injuries [15].

These conclusions are in agreement with our analyses of the relative level of K18 phosphorylation in livers classified according to ALT activities (Fig. 3). While phosphorylation of Ser52 increased in livers with elevated ALT (≥ 40 U/L) (Fig. 3C), phosphorylation of Ser33 was observed in HBV infected livers regardless of ALT activities (Fig. 3B). To our knowledge, this is the first time that K18 phosphorylation was directly compared with ALT activities in diseased livers. The association of Ser33 phosphorylation with HBV infection was further demonstrated in the immunohistological analyses of the liver sections of an inactive HBV carrier (Fig. 4). A high level of Ser33 phosphorylation was detected in only hepatocytes infected with HBV, as indicated by the strong HBsAg staining (Fig. 4A and 4A' cf. Fig. 4B and 4B'). On the other hand, we observed merely basal level of Ser52 phosphorylation in the hepatocytes of the carriers regardless of HBsAg staining (Fig. 4C and 4C' cf. Fig. 4D and 4D'). Toivola and colleagues observed increased phosphorylation of both Ser33 and Ser52 in livers from patients with chronic non-cirrhotic HCV and cirrhosis [16]. The different cytochemistry and molecular events induced by HBV and HCV could attribute to the difference in Ser52 phosphorylation. Nevertheless, the same authors also observed limited reorganization of the keratin filament networks in pre-cirrhotic HCV livers [17]. The absence of keratin and the destruction of cytoskeletal networks are the hallmarks of hepatocytic injuries [30]. If limited reorganization of the keratin filament networks is also an indication of limited injuries in HCV livers, the basal level phosphorylation of Ser52 in non-cirrhotic HBV livers with relative low ALT activities (≤ 40 U/L) may indicate that in these hepatocytes viral infection does not cause injuries that trigger Ser52 phosphorylation and related cellular protection. If this is indeed true, phosphorylation of Ser33 in K18 could serve as an early indication of HBV infection.

In summary, phosphorylation of Ser33 and Ser52 in K18 may serve as reliable progression markers of chronic hepatitis B. Although it remains to be determined the precise molecular mechanism through which HBV alters K18 phosphorylation, specifically how differential phosphorylation of Ser33 and Ser52 is modulated in hepatocytes at different stages of the liver disease, our study suggests that the two phosphorylation events may underlie different hepatocytic events associated with HBV.

## Conclusion

In conclusion, K18 phosphorylation is a progression marker for CHB. K18 Ser52 phosphorylation is associated with the cellular protection in hepatocytes with HBV infection, while phosphorylation of Ser33 in K18 may serve as an early indication of HBV infection.

## Methods

### Antibodies

The anit-K18 monoclonal antibody and the phospho-specific anti-K18 Ser33 and -Ser52 polyclonal antibodies were from Santa Cruz Biotechnology (Santa Cruz, CA). Anti-HBsAg monoclonal antibody and anti-tubulin monolonal antibody were from Sigma-Aldrich Co (Eugene, OR).

### Patients

Hepatitis B e-antigen (HBeAg)-negative chronic HBV infection was diagnosed based on positive hepatitis B surface antigen (HBsAg) and negative HBeAg for ≥ 6 months. Patients with HBeAg-negative chronic HBV infection, persistently normal ALT activity for ≥ 12 months (determined at least every 3 months during the first year and at least every 6 months thereafter) and baseline serum HBV DNA below 20,000 IU/ml were considered as inactive chronic carriers. ALT and AST were detected by the Hitachi 7600 Series automatic biochemical analyzer (Hitachi, Tokyo, Japan).

A total of 61 HBsAg positive patients were recruited from October, 2006 to September, 2008 at You'an Hospital, Capital Medical University (Beijing, China). They were divided into two groups, 40 patients with CHB and 21 with liver cirrhosis (LC, active or inactive [27]). Normal control liver tissues were from donors undergone liver transplantation. Those with CHB were obtained by liver centesis. Cirrhosis specimens were obtained from transplant programs. This study was approved by the local ethics committee, and informed consent was obtained from each patient.

### Histological analysis

Sections of liver biopsy specimens were stained with hematoxylin-eosin, and assessed by a pathologist without prior knowledge of the clinical and virological results. The histological changes were classified according to Ishak et al [28].

### Immunoblot analysis

Liver biopsies or explants were snap-frozen in liquid nitrogen and kept at -80°C until use. The explant pieces or liver centesis tissues, of approximately 0.5 cm^3^, were homogenized in 500 μl and 30 μl, respectively, of high salt lysis buffer (150 mM NaCl, 1% NP-40, 0.5% deoxycholate, 0.1% SDS, 50 mM Tris [pH 8.0], 5 mM EDTA) with protease inhibitors (10 μg/ml PMSF). Total cellular lysates were separated on 12% SDS-PAGE, and then transferred to PVDF membrane. Following the standard protocol, the protein blots was blocked with 5% nonfat milk, probed sequentially with specific primary antibodies and horseradish peroxidase-conjugated secondary antibodies. The detection of specific proteins on blots was achieved with enhanced chemiluminescence (Pierce SuperSignal, Thermo Fisher Scientific Inc. Rockford, IL) captured on X-ray films. The relevant bands were quantified with a Bio-Rad scanning densitometer (GS-710) using Quantity One software (Bio-Rad Laboratories Inc, version 4.62, Hercules, CA). K18 signal was used to normalize the relative level of phosphorylation of Ser33 and Ser52.

### Immunofluorescence microscopy

Frozen sections of fresh liver tissues were prepared using the standard techniques. They were fixed with 10% formaldehyde/PBS, incubated in 1% Triton X-100/PBS for 10 min, blocked with 3% BSA/PBS, and probed with mouse anti-HBsAg mAb, for which tetramethyl rhodamine isothiocyanate (TRITC)-conjugated secondary antibodies were used. Slices were then probed with anti-K18 Ser52 or -Ser33 antibodies, for which fluorescent isothiocyanate (FITC)-conjugated second secondary antibodies were used. Nuclei were counterstained with 4,6-diamidino-2-phenylindole (DAPI). Sections were examined with a fluorescence microscope (Nikon Eclipse 80i). Figures were generated with Adobe Photoshop (Adobe Systems Inc, version 7.0, San Jose, CA).

### Statistical analysis

Statistical analyses of the results were performed by Student Newman Keuls Test (SNK) and Least Significant Difference Procedure (LSD) with an analysis software, Statistical Package for Social Science (SPSS Inc. version 11.5, Chicago, IL), and *P *< 0.05 was considered statistically significant.

## Abbreviations used in this paper

(HBV): Hepatitis B virus; (CHB): Chronic hepatitis B; (K18): Keratin 18.

## Competing interests

The authors declare that they have no competing interests.

## Authors' contributions

YS was responsible for Western Blotting analysis, interpretation, and writing of this manuscript, SHS was involved in data analysis and drafting the manuscript, YLL carried out immunofluorescence analysis, JFL and TZ coordinated sample collection, HW and XYC revised the manuscript, DXC and YSZ were the principal investigator and were primarily responsible for all aspects of the funding. All authors read and approved the final version.

## References

[B1] Omary MB Ku NO Toivola DM Keratins, Guardians of the liver Hepatology 2002 35 251 257 10.1053/jhep.2002.31165 11826396

[B2] Coulombe PA Omary MB 'Hard' and 'soft' principles defining the structure, function and regulation of keratin intermediate filaments Curr Opin Cell Biol 2002 14 110 122 10.1016/S0955-0674(01)00301-5 11792552

[B3] Marceau N Loranger A Gilbert S Daigle N Champetier S Keratin mediated resistance to stress and apoptosis in simple epithelial cells in relation to health and disease Biochem Cel Biol 2001 79 543 555 10.1139/bcb-79-5-543 11716296

[B4] Gonzalez-Quintela A Abdulkader I Campos J Fernandez-Hernandez L Lojo S Serum levels of keratin-18 fragments [tissue polypeptide-specific antigen (TPS)] are correlated with hepatocyte apoptosis in alcoholic hepatitis Dig Dis Sci 2009 54 648 653 10.1007/s10620-008-0371-2 18618253

[B5] Gonzalez-Quintela A Lojo S Otero E Perez LF Keratin-18 as a marker of steatohepatitis Hepatology 2006 44 27 33 17256723 10.1002/hep.21550

[B6] Yilmaz Y Dolar E Ulukaya E Akgoz S Keskin M Kiyici M Aker S Yilmaztepe A Gurel S Gulten M Nak SG Soluble forms of extracellular cytokeratin 18 may differentiate simple steatosis from nonalcoholic steatohepatitis World J Gastroenterol 2007 13 837 844 17352011 10.3748/wjg.v13.i6.837 PMC4065917

[B7] Simopoulos C Tsaroucha AK Asimakopoulos B Giatromanolaki A Gavriilidis P Polychronidis A Karaiannakis A Total and caspase-cleaved cytokeratin 18 in chronic cholecystitis: A prospective study BMC Gastroenterol 2008 8 14 18 10.1186/1471-230X-8-14 18460214 PMC2396167

[B8] Papatheodoridis GV Hadziyannis E Tsochatzis E Chrysanthos N Georgiou A Kafiri G Manolakopoulos S Tiniakos DG Giannousis I Manesis EK Archimandritis AJ Serum apoptotic caspase activity as a marker of severity in HBeAg-negative chronic hepatitis B virus infection Gut 2008 57 500 506 10.1136/gut.2007.123943 18025069

[B9] Caulin C Ware CF Magin TM Oshima RG Keratin-dependent, epithelial resistance to tumor necrosis factor-induced apoptosis J Cell Biol 2000 149 17 22 10.1083/jcb.149.1.17 10747083 PMC2175089

[B10] Gilbert S Loranger A Daigle N Marceau N Simple epithelium keratins 8 and 18 provide resistance to Fas-mediated apoptosis. The protection occurs through a receptor-targeting modulation J Cell Biol 2001 154 763 773 10.1083/jcb.200102130 11514590 PMC2196458

[B11] Ku NO Soetikno RM Omary MB Keratin mutation in transgenic mice predisposes to Fas but not TNF-induced apoptosis and massive liver injury Hepatology 2003 37 1006 1014 10.1053/jhep.2003.50181 12717381

[B12] Inada H Izawa I Nishizawa M Fujita E Kiyono T Takahashi T Momoi T Inagaki M Keratin attenuates tumor necrosis factor-induced cytotoxicity through association with TRADD J Cell Biol 2001 155 415 426 10.1083/jcb.200103078 11684708 PMC2150850

[B13] Ku NO Michie S Resurreccion EZ Broome RL Omary MB Keratin binding to 14-3-3 proteins modulates keratin filaments and hepatocyte mitotic progression Proc Natl Acad Sci USA 2002 99 4373 4378 10.1073/pnas.072624299 11917136 PMC123655

[B14] Galarneau L Loranger A Gilbert S Marceau N Keratins modulate hepatic cell adhesion, size and G1/S transition Exp Cell Res 2007 313 179 194 10.1016/j.yexcr.2006.10.007 17112511

[B15] Ku NO Michie SA Soetikno RM Resurreccion EZ Broome RL Omary MB Mutation of a major keratin phosphorylation site predisposes to hepatotoxic injury in transgenic mice J Cell Biol 1998 143 2023 2032 10.1083/jcb.143.7.2023 9864372 PMC2175212

[B16] Omary MB Ku NO Tao GZ Toivola DM Liao J 'Heads and tails' of intermediate filament phosphorylation: multiple sites and functional insights Trends Biochem Sci 2006 31 383 394 10.1016/j.tibs.2006.05.008 16782342

[B17] Toivola DM Ku NO Resurreccion EZ Nelson DR Wright TL Omary MB Keratin 8 and 18 hyperphosphorylation is a marker of progression of human liver disease Hepatology 2004 40 459 466 10.1002/hep.20277 15368451

[B18] Stumptner C Omary MB Fickert P Denk H Zatloukal K Hepatocyte cytokeratins are hyperphosphorylated at multiple sites in human alcoholic hepatitis and in a mallory body mouse model Am J Pathol 2000 156 77 90 10623656 10.1016/S0002-9440(10)64708-6 PMC1868635

[B19] Denk H Stumptner C Zatloukal K Mallory bodies revisited J Hepatol 2000 32 689 702 10.1016/S0168-8278(00)80233-0 10782920

[B20] Zatloukal K Stumptner C Fuchsbichler A Peter F Carolin L Michael T Helmut D The keratin cytoskeleton in liver diseases J Pathol 2004 204 367 376 10.1002/path.1649 15495250

[B21] Fickert P Trauner M Fuchsbichler A Stumptner C Zatloukal K Denk H Mallory body formation in primary biliary cirrhosis is associated with increased amounts and abnormal phosphorylation and ubiquitination of cytokeratins J Hepatol 2003 38 387 394 10.1016/S0168-8278(02)00439-7 12663227

[B22] Fausther M Villeneuve L Cadrin M Heat shock protein 70 expression, keratin phosphorylation and Mallory body formation in hepatocytes from griseofulvin-intoxicated mice Comp Hepatol 2004 3 5 21 10.1186/1476-5926-3-5 15307891 PMC516018

[B23] Koziel MJ Cellular immune responses against hepatitis C virus Clin Infect Dis 2005 41 Suppl 1 25 31 10.1086/429492 16265610

[B24] Isabel F Dysregulation of apoptosis in hepatocellular carcinoma cells World J Gastroenterol 2009 15 513 520 10.3748/wjg.15.513 19195051 PMC2653340

[B25] Schuchmann M Galle PR Apoptosis in liver disease Eur J Gastroenterol Hepatol 2001 13 785 790 10.1097/00042737-200107000-00005 11474307

[B26] Staib F Hussain SP Hofseth LJ Wang XW Harris CC TP53 and liver carcinogenesis Hum Mutat 2003 21 201 216 10.1002/humu.10176 12619106

[B27] Chinese Medical Association Infectious and Parasitic Disease, and Hepatology Branches, Prevention and treatment of viral hepatitis Chin J Hepatol 2000 8 324 329

[B28] Ishak K Baptista A Bianchi L Callea F De Groote J Gudat F Denk H Desmet V Korb G MacSween RN Phillips MJ Portmannl BG Paulsen H Scheuer PJ Schmid M Thaler H Histological grading and staging of chronic hepatitis J Hepatol 1995 22 696 699 10.1016/0168-8278(95)80226-6 7560864

[B29] Ku NO Liao J Chou CF Omary MB Implications of intermediate filament protein phosphorylation Cancer Metastasis Rev 1996 15 429 444 10.1007/BF00054011 9034602

[B30] Bert S Mieke H Wendy K Maartje B Stephan M Mathie PGL Marius N Viveka B Peter B Bertil B Lane EB Omary MB Hans J Frans CSR Keratin 8/18 breakdown and reorganization during apoptosis Exp Cell Res 2004 297 11 26 10.1016/j.yexcr.2004.02.019 15194421

